# Phylogenetic Studies of the Three RNA Silencing Suppressor Genes of South American CTV Isolates Reveal the Circulation of a Novel Genetic Lineage

**DOI:** 10.3390/v7072814

**Published:** 2015-07-22

**Authors:** María José Benítez-Galeano, Leticia Rubio, Ana Bertalmío, Diego Maeso, Fernando Rivas, Rodney Colina

**Affiliations:** 1 Laboratorio de Virología Molecular, Centro Universitario Regional Noroeste (CENUR Noroeste), Universidad de la Republica (UdelaR), Rivera 1350, 50000 Salto, Uruguay; E-Mail: mbenitezgaleano@gmail.com; 2 Programa Nacional de Investigación en Producción Citrícola, Instituto Nacional de Investigación Agropecuaria (INIA), Urguay; E-Mails: lrubio@inia.org.uy (L.R.); abertalmio@inia.org.uy (A.B.); dmaeso@inia.org.uy (D.M.); cfrivas@inia.org.uy (F.R.)

**Keywords:** *Citrus Tristeza Virus*, RT-PCR, RNA Silencing Suppressor Genes, Phylogeny, Recombination, Novel Genetic Lineage, Uruguay

## Abstract

*Citrus Tristeza Virus* (CTV) is the most economically important virus of citrus worldwide. Genetic diversity and population structure of CTV isolates from all citrus growing areas from Uruguay were analyzed by RT-PCR and cloning of the three RNA silencing suppressor genes (p25, p20 and p23). Bayesian phylogenetic analysis revealed the circulation of three known genotypes (VT, T3, T36) in the country, and the presence of a new genetic lineage composed by isolates from around the world, mainly from South America. Nucleotide and amino acid identity values for this new genetic lineage were both higher than 97% for the three analyzed regions. Due to incongruent phylogenetic relationships, recombination analysis was performed using Genetic Algorithms for Recombination Detection (GARD) and SimPlot software. Recombination events between previously described CTV isolates were detected. High intra-sample variation was found, confirming the co-existence of different genotypes into the same plant. This is the first report describing: (1) the genetic diversity of Uruguayan CTV isolates circulating in the country and (2) the circulation of a novel CTV genetic lineage, highly present in the South American region. This information may provide assistance to develop an effective cross-protection program.

## 1. Introduction

Citrus is one of the most important commercial fruit crop worldwide. *Citrus Tristeza Virus* (CTV) is the most serious viral pathogen of citrus and has been responsible for the loss of over 100 million trees in the past 70 years [1]. CTV is semi-persistently transmitted by several aphid species, specially *Toxoptera citricida* and *Aphis gossypii*, the former being the most efficient in virus transmission [2]. It is also graft transmitted and is limited to the phloem of infected plants species of *Rutaceae* [3]. Isolates of CTV are traditionally distinguished by the production of three different symptoms: (i) decline of citrus species grafted on sour orange rootstock; (ii) stunting, stem pitting and poor yield of different citrus varieties regardless of the rootstock used; and (iii) yellowing and growth cessation of sour orange, lemon or grapefruit seedlings, of which the first two are significant problems for the industry [1].

CTV virions (*Closteroviridae*, *Closterovirus*) are flexuous filaments of 2000 × 11 nanometers in size, with a helical nucleocapsid architecture consisting of two coat proteins. One of these proteins is a 25 kDa major capsid protein (CP), which forms a long virion body covering 95% of the particle length and is encoded by the p25 gene [4,5,6]. The positive sense, single stranded RNA genome of 19.3 kb in size, the largest reported for plant viruses so far, contains 12 open reading frames (ORFs) encoding at least 19 proteins [5,7]. The two 5′-proximal ORFs encoding components of the replicase complex are translated directly from the genomic RNA, while the remaining ORFs expressed via ten 3′ co-terminal subgenomic RNAs (sgRNAs) and differ in their accumulation and time course appearance during the infection process [8,9]. ORF1 expresses one large polyprotein (ORF1a), as well as the RNA-dependent RNA-polymerase (ORF1b) via +1 frameshift [4]. The ten other ORFs encode the major and minor coat proteins (p25 and p27), three suppressors of RNA silencing (p25, p20 and p23), two genes expressing a heat shock protein homolog (p65) and a protein with a diverged coat protein motif, both required for virion assembly, and three proposed host range genes (p33, p13, and p18) [10,11,12]. Additionally, p20 accumulates in amorphous inclusion bodies and p23, an RNA-binding protein, controls asymmetrical accumulation of plus and minus strands during RNA replication and is involved in symptom expression [13,14,15,16].

As a result of error-prone replication, recombination events, and repeated vector-mediated transmission, naturally occurring CTV isolates exist as a population of related sequence variants displaying different biological characteristics [17,18]. In this matter, studies of CTV population genetic diversity may be highly relevant to develop viral control strategies, such as cross protection programs, which have been successfully developed in countries, such as Brazil, South Africa, or Peru [19]. The recipe for cross-protection to work is, first, identify the severe isolates that need to be controlled, and then find mild isolates of these genotypes to cross-protect the plants [20].

During the last few years, CTV genetic diversity was studied using different molecular techniques, including RT-PCR and phylogenetic analysis. Nevertheless, there has been no consensus among researchers to analyze the same regions of the genome, and this led to different classifications systems [8,21,22,23,24,25]. Recently, Harper proposed a new typing system based on phylogenetic relationships using complete genomes that groups CTV isolates in six distinct genotypes [26]. The author also proposes an alternative way, based on the amplification of segments from the 5′ half region of the genome, with the aim to standardize a CTV typing criteria [26].

Few studies describing CTV genetic diversity circulating in South America have been carried out [27,28,29]. Iglesias and co-workers described the genetic variation of three genomic regions from CTV isolates circulating in the two main citrus growing areas of Argentina [30]. Based on the typing system available at that time, they found isolates grouping with the VT severe strain, as well as isolates similar to the mild reference strain, T30, showing a high genetic diversity of CTV isolates present in the country [30]. Nevertheless, CTV genetic diversity has been growing in the last few years, as well as the typing systems [24,25,26].

In Uruguay, citrus is one of the main fruit crops and it is distributed throughout the country on about 17,000 Ha with a total annual production of 270,000 tons. The most representative species are sweet oranges (Navel and Valencia groups), followed by mandarins and lemons. Almost 90% of the trees in the country are grafted onto *Poncirus trifoliata* rootstock. The majority of the citrus orchards are located in the northwestern region (Salto and Paysandú provinces). It should be noted that Salto and Concordia are the main soft citrus growing areas of Uruguay and Argentina, respectively, and they are located 10 km away from each other, separated by the Uruguay River. Studies performed in 1955, showed that CTV is endemic and its most efficient vector, *T. citricida,* is present in the country and the region [31].

Considering that the Uruguayan citrus industry is experiencing a great expansion, looking for better productivity and fruit quality for fresh fruit markets, and taking into account that there is no data about the genetic diversity of CTV in Uruguay, it is necessary to generate this information with the aim to perform a long term cross protection program in the country. With this aim, we analyzed the genetic diversity of CTV isolates obtained from different geographic regions of the country using RT-PCR and the cloning of the three RNA silencing suppressor genes, p25, p20, and p23. Next, in order to characterize the genetic variability of CTV, we performed an extensive Bayesian phylogenetic analysis, comparing the Uruguayan sequences with isolates from all over the world. We also analyzed the presence of recombinant strains. In summary, our study demonstrates the presence of severe genotypes VT and T3, and the intermediate genotype T36, in Uruguay. Surprisingly, we found a new genetic lineage tentatively named NC (New Clade), formed by isolates from the countries of five continents, mainly South America. To our knowledge, this is the first study, which describes the genetic variability and population structure of CTV isolates circulating in Uruguay, based on molecular phylogenetic analysis of the three RNA silencing suppressor genes and also the first report describing the existence of a new genetic lineage of CTV, strongly present in the South American region.

## 2. Results

### 2.1. Population Structure and Geographical Distribution of CTV Isolates in Uruguay

Given the co-circulation of different CTV genotypes and the possibility of re-infection caused by the virus vector, we analyze the DNA clones obtained from the Uruguayan isolates, in order to know a detailed composition of the population structure. To achieve this, we evaluate 331 DNA clones of three genome regions of thirteen CTV isolates, establishing phylogenetic relationships between them.

**Figure 1:**
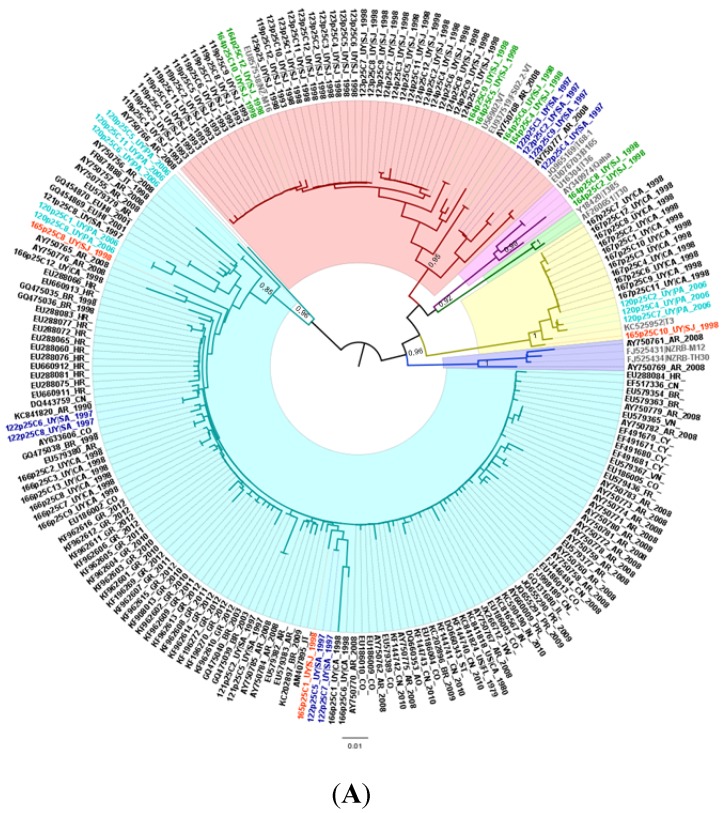

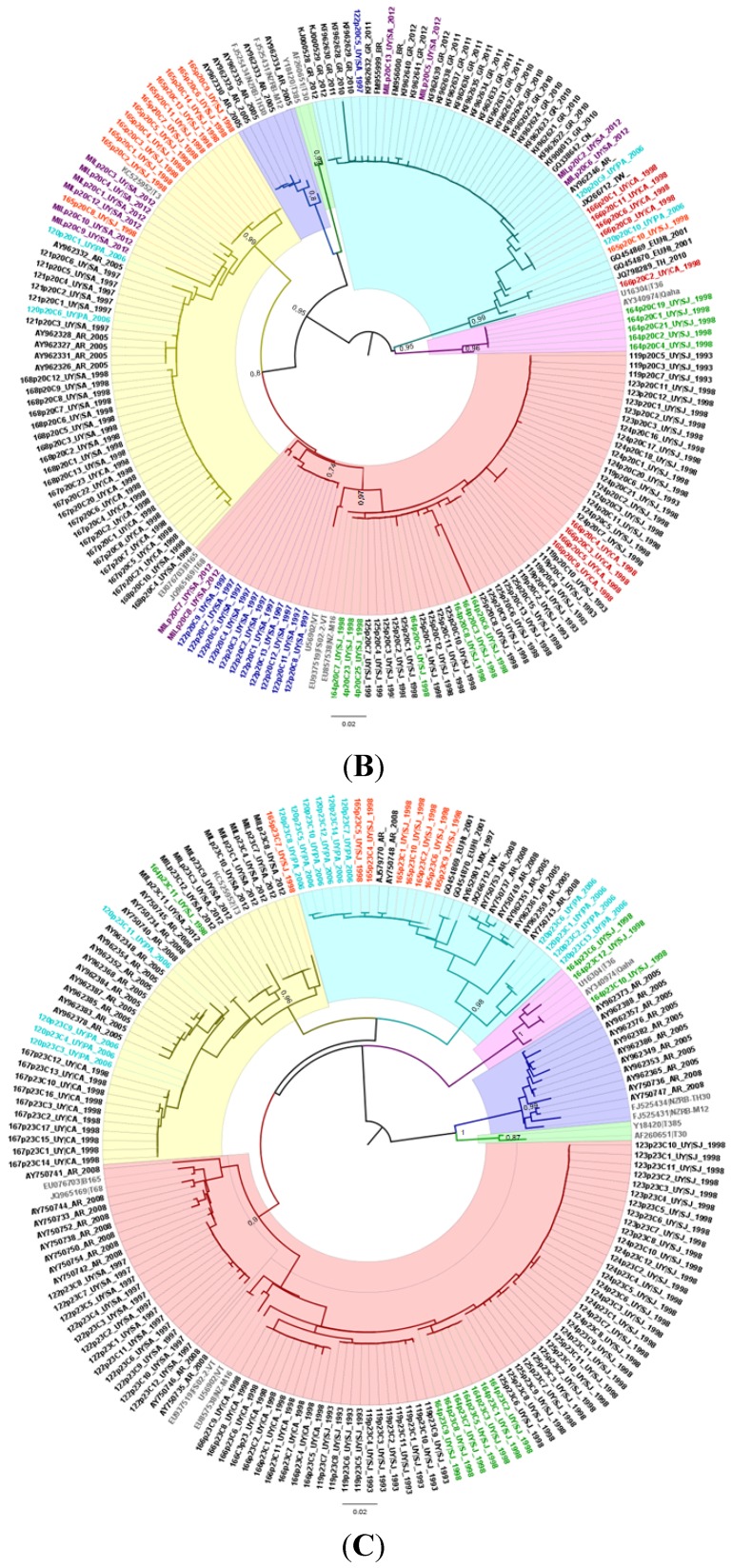
Phylogenetic trees for CTV p25 (**A**); p20 (**B**) and p23 (**C**) genes. Colored and highlighted branches represent genotypes: T30 (green), RB (blue), T36 (violet), VT (red), T3 (yellow) and NC (turquoise). Principal node aLRT values are indicated. Reference strains are marked in grey. Uruguayan samples composed by heterogeneous populations are differentially colored.

Phylogenetic analysis of the three regions sequenced in this study indicated the presence of a new genetic lineage (named NC by the authors), different from all major extant CTV genotypes, VT, T36, T30, RB, T68, and T3, and supported by high aLRT values (Figure 1). This new genetic lineage is formed by sequences from Uruguay and other geographic regions from all over the world. On the other hand, genotypes VT, T3, T30, T36, and RB are clearly defined in the phylogenies made for the three regions under study. Nevertheless, genotype T68 was not observed as a monophyletic lineage at any case, using the three regions analyzed in the present study.

In order to provide geographical information on the distribution of the different CTV genotypes in Uruguay, the variations of the p20 gene were calculated for both citrus growing areas of the country (Figure S1. Genotype geographic distribution of p20 gene). VT genotype was prevalent in the southern region, while T3 was predominant in the north region of the country. The VT genotype was present in 28% of the sequenced DNA clones and the NC genotype was represented by 15% of the DNA clones in the northern region of the country Whereas T3, NC, and T36 genotypes were present by 27%, 7%, and 6% of DNA clones, respectively, in the southern citrus growing area of Uruguay.

To build p25 gene phylogenetic tree, a total of 214 sequences of 604 bp in length were used. The dataset was composed by 86 Uruguayan isolates obtained in the present study, plus 12 reference sequences (see *Materials and Methods*), 31 Argentinean isolates and 85 isolates obtained from GenBank by BLAST for comparing purposes [30]. Tree topology shows six well defined groups (Figure 1A). Uruguayan isolates were grouped with genotypes VT (54%), T3 (17%) and T36 (2%). Surprisingly, 27% of the isolates obtained in this work grouped into the NC clade together with isolates from Argentina, Brazil, Colombia, Venezuela, Portugal, India, China, Angola, Greece, Taiwan, and USA. None of the Uruguayan isolates grouped within the T30 or RB genotypes.

In the case of the p20 gene phylogenetic tree, the dataset had an amount of 182 sequences of 427 bp in length. In this case, 130 Uruguayan isolates from this study, as well as 12 reference strains, 10 Argentinean isolates, and 30 sequences retrieved from GenBank by BLAST, were used (Figure 1B) [32]. As for the p25 gene, VT (48%), T3 (38%), and T36 (4%) genotypes were observed within the Uruguayan isolates, and the NC clade grouped 10% of the Uruguayan sequences and isolates from Argentina, Greece, Spain, China, Thailand, Taiwan, and USA.

Finally, the phylogenetic analysis of the p23 gene showed the Uruguayan isolates grouping into the same genotypes as for the other two genes with frequencies of 60% for VT, 22% for T3, 3% for T36, and 15% for NC clade, which, in this case, was composed by isolates from Uruguay, Argentina, USA, Mexico, and Taiwan (Figure 1C). In this analysis we use a dataset of 174 sequences of 557 bp in length, with 115 DNA clones sequences from Uruguayan isolates, 42 Argentinean isolates from Iglesias and co-workers, 5 sequences retrieved from GenBank by BLAST, as well as the 12 reference strains [30,32].

For the three regions analyzed, heterogeneous populations were found in different Uruguayan samples, such as 120, 122, 164, 165, 166, and MIL, which means that clones analyzed for each sample grouped into different genotypes (Figure 1). Samples 120, 164, and 165 presented sequences of at least two genotypes for the three regions analyzed. The most variable region, or the region with more heterogeneous sequence variants, was p20, in which we found clones grouping into the four described genotypes in half of the samples (Figure 1). Interestingly, field sample MIL was composed of clones of genotypes VT, T36, and NC in the p20 region (Figure 1B), as well as sample 164 in region p23, composed by clones of genotypes T3, VT, and T36 (Figure 1C).

Inversely, samples 119, 123, 124, 125, and 167 were shown to be homogeneous, which means that all the clones examined for the three analyzed genes were from the same genotype, being the first four of the VT genotype and the last one of the T3 genotype. In the case of sample 168, it was only possible to clone the p20 fragment, and all the analyzed clones correspond to the T3 genotype.

### 2.2. Genetic Divergence Analysis

In order to know the nucleotidic and amino acidic identities of the new monophyletic NC genetic lineage, and the differences compared to extant genotypes as well, intra- and inter-genotype mean distances were estimated using MEGA 6 based on the Kimura 2 parameter model [33]. In general, genetic identities between NC genetic lineage and different genotypes (inter-genotype nucleotide diversity) were lower than the NC average (intra-genotype diversity) (Table 1). Nucleotidic identity values for the NC genetic lineage ranged from 98.7% to 98.9% for the p25 gene, 98.7% to 99.1% for the p20 gene and 97.2% to 97.8% for the p23 gene, showing the latter at the lowest values (Table 1). Regarding amino acidic identity values, which were similar to the nucleotidic ones, they ranged from 98.6% to 99.0%, 98.2% to 99.4% and 96.2% to 97.6% for p25, p20 and p23, respectively. The lowest identity intra-NC clade value was observed in the p23 gene, both at the nucleotide and amino acid level (Table 1). Finally, the NC genetic lineage shows a closer relationship with some specific genotypes in two of the studied genomic regions. In the p25 gene, the NC genetic lineage was more similar to the RB genotype at the nucleotide level, and to the T3 genotype at the aminoacid level. In the case of the p20 region, NC genetic lineage shows similarity with the T36 genotype at both levels, whereas the p23 region was T3-like and T30-like at the nucleotide and aminoacid levels, respectively (Table 1).

**Table 1:** Averaged nucleotide and amino acid identities for each region of the NC lineage and the comparison with other extant genotypes.

Region	NC average		RB		T3		T30		T36		VT	
	nt	aa	nt	aa	nt	aa	nt	aa	nt	aa	nt	aa
**p25**	98.8 ± 0.1	98.8 ± 0.2	**90.6 ± 1.3**	95.2 ± 1.2	89.4 ± 1.5	**96.4 ± 1.2**	89.1 ± 1.5	94.4 ± 1.5	89.1 ± 1.5	93.6 ± 1.6	90.2 ± 1.4	95.4 ± 1.2
**p20**	98.9 ± 0.2	99.1 ± 0.3	84.4 ± 2.5	93.9 ± 2.1	82.1 ± 2.7	92.6 ± 2.2	81.8 ± 2.9	92.2 ± 2.4	**88.6± 1.8**	**95.3 ± 1.8**	84.1 ± 2.4	93.1 ± 2.2
**p20**	97.5 ± 0.3	96.9 ± 0.7	87.3 ± 1.5	88.2 ± 2.2	**90.5 ± 1.2**	89.9 ± 2.0	89.9 ± 1.2	**90.9 ± 2.0**	89.3 ± 1.3	88.5 ± 2.3	89.3 ± 1.3	88.7 ± 2.1

Bold numbers indicates the genotype with greatest identity for each region.

### 2.3. Recombination Analysis

**Figure 2:**
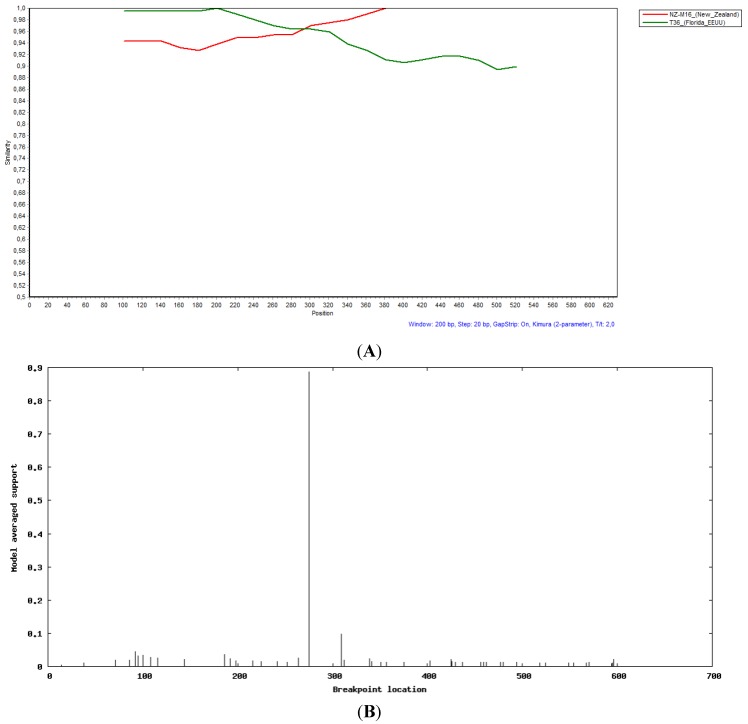
Evidence of recombination of clone 6 from sample 164 on p25 region between NZ-M16 and T36 isolates (**A**); Recombination point location detected by GARD tool (**B**).

Due to the incongruities shown by sample 164 on the p25 gene phylogenetic tree, we decided to perform recombination studies on this sample. We conducted the analysis on the three genes using Simplot and GARD programs [34,35]. No evidence of recombination in the p20 and p23 analyzed regions was found. However, as shown in Figure 2, evidence of recombination in clones 4 and 6 of this sample was found in the p25 gene. Simplot analysis showed that the recombination event was produced between NZ-M16-like and T36-like strains, and the recombination point, determined by GARD, was at position 274 of the fragment corresponding to position 16484 of the genome, based on strain U16304 (Figure 2).

## 3. Discussion

### 3.1. Composition of Viral Populations and Geographical Information

In the matter of sequence variants in the composition of the viral populations, the presence of genomes from different genotypes in the same plant reinforces the fact that one tree could be co-infected by more than one genotype. This is explained by the high frequency of vector-mediated re-infections during tree life, which is expected to occur over several decades, and the presence of the most efficient vector, *T. citricida*, which is endemic in the country [30,36]. This fact could also explain the great variability observed in the MIL sample, which was collected almost ten years after the rest of the samples, thus tree exposure to the vector was higher. Nevertheless, this result, derived from an individual sample, remains insufficient to draw a conclusive statement. Henceforth, we propose the analysis of a greater number of field samples, collected up until recently.

Taking into account that both citrus growing areas in Uruguay are represented in our sampling, the distribution of genotypes reported in the present work, despite the small amount of samples, are representative and present a larger picture of the genotypes present in the country. This is the first report of CTV genetic variation in the country and this information could be used as a baseline of future temporal and spatial spreads of CTV isolates in Uruguay.

### 3.2. Phylogenetic Relationships among the Isolates

The genomic sequences indicate that the Uruguayan isolates are members of three extant CTV phylogenetic lineages, VT, T3, and T36. Additionally, 10% to 27% of these isolates were found in the NC monophyletic group. This genetic lineage was previously described by other authors, although with different names, such as group 5 based on Nolasco’s classification system or group 7 in Wang and co-workers’ article [24,37,38,39,40,41]. Because of its high genetic conservation, all of the aforementioned reports are based on the study of the p25 gene, which is widely used to classify CTV [42]. Two aspects deserve to be highlighted. First, many of the sequences retrieved from GenBank by BLAST and clustering within the NC genetic lineage are the same as those found in group 5 or group 7, revealing that a divergent group exists compared with the rest of the extant CTV genotypes. Second, isolate HA16-5, which was not included into any of the existing genotypes, and suggested by Harper as a future possible new genotype, interestingly grouped into this NC genetic lineage for the three analyzed regions in the present work [26].

Another sequence that supports the topologies of the phylogenetic trees obtained in this work, for all three analyzed regions, is the recently sequenced complete genome of a Taiwanese strain (JX266713). We performed complete genomes phylogenies with Maximum Likelihood (ML) and Maximum Parsimony (MP) methods and this sequence from Taiwan grouped with the HA16-5 isolate from Hawaii, suggesting a new genotype for CTV (Figure S2. Complete genomes phylogenetic trees). Furthermore, the analysis of the three regions, separately, shows HA16-5 and Taiwan clustering together, forming this NC monophyletic group. Although no complete genomes were used in the present work, the consistency of information between the three analyzed regions is clear.

It is worth noticing that genotype T68 does not appear in the p25, p20, and p23 genes phylogenies. This could be explained by the fact that isolates from this lineage are recombinant, showing a VT-like 3′half part in their genomes [26]. Nevertheless, the robustness of the results presented here has been probed by means of phylogenetic trees topologies. For all analyzed regions, the phylogenetic relationships between genotypes remain similar and every single node is supported by high aLRT values. Although it has been suggested the amplification of multiple sites of ORF1a/1b to type CTV, it will take time for researchers from different parts of the world to accept and apply this methodology to reach uniformity in CTV typing criteria [26].

According to data reported in Brazil, Argentina, and other parts of the world, the VT genotype was predominant within CTV variants circulating in Uruguay, supporting the prevalent status of the genotype worldwide and reinforcing results obtained in the present work [30,32,42,43,44]. Previous phylogenetic studies performed with Argentinean isolates showed the circulation of different CTV genotypes in the country [30]. However, based on the typing system used, some of the isolates could not be clearly assigned to a specific genotype [30]. Nevertheless, using the new typing system proposed by Harper [26], we could assign genotypes to all these Argentinean isolates and, surprisingly, it turned out that many of these sequences grouped into the NC genetic lineage reported here. These results confirm that the NC genetic lineage has been present in the South American region for some decades.

### 3.3. Genetic Divergence of CTV Populations in Uruguay

Comparative analyses of the p25, p20, and p23 regions of CTV-NC genetic lineage members with other CTV genotypes isolates provided evidence that this monophyletic group is different from the previously known and very well described genotypes. The three regions analyzed showed less than 92% sequence identity with other reported CTV genotypes, whereas, on the other hand, sequence identity within the NC monophyletic group was considerably higher, with values up to 99%. As was expected, the p23 region shows the lowest identity values within and between groups. This gene is not only responsible for RNA interference silencing suppression, but also controls negative strand accumulation, thus, diversification of p23 is predictable, as both host antiviral RNAi genes and viral suppressors of silencing are known to rapidly evolve [45].

### 3.4. Evidence of Recombination

Recombination has played a significant role in the evolution of all the known CTV genotypes [18,46]. Evidence of multiple recombination events has been widely described through CTV evolutionary history [18,43]. This is to be expected due to the frequent mixtures of two or more strains in the same plant, and also because of the efficient vector transmission. The fact that only two clones of the eight analyzed for sample 164 on the p25 region were recombinant shows the co-existence of recombinant and non-recombinant isolates in the plant at the same time. Nevertheless, we could not find the parental sequences involved in the recombination event, suggesting the divergence of the recombinant over the years.

## 4. Materials and Methods

### 4.1. Virus Isolates

Thirteen CTV isolates were collected almost 20 years ago from naturally-infected field trees in different citrus-growing areas of Uruguay, and were maintained in rough lemon (*Citrus jambhiri*) plants in insect-proof greenhouses (Table 2). CTV and *T. citricida* are endemic in these regions since at least 1955, thus, the plants were probably infected since their introduction in the field. Symptom evaluation was performed for these isolates in graft-inoculated Mexican lime (*Citrus aurantifolia*) plants. After a year from inoculation, the bark from every branch was peeled off looking for stem pitting symptoms (SP). In 1999, serological analysis, such as ELISA, was performed on the isolates. Geographical distribution, presence or absence of SP in Mexican lime, collection date, and ELISA test result are summarized in Table 1. These isolates belong to the collection of the National Institute of Agricultural Research (INIA), Salto Grande, Uruguay.

The sample named MIL was collected from the field in 2012 and no ELISA test or any symptom evaluation analysis was performed (Table 2). After collection, it was immediately processed in the same way as the other samples.

**Table 2:** Characteristics of the Uruguayan isolates used in the present study.

Isolate	Host	Origin	Collection date	Aspect of the tree in the field	Stem Pitting	ELISA	Number of clones		
							p25	p20	p23
119	Lemon	San José	1993	Stunted	NA	+	12	9	11
120	Washington Navel sweet orange	Paysandú	2006	Stunted	NA	+	8	4	14
121	Star Ruby grapefruit	Salto	1997	Stunted	+	-	3	6	NC
122	Star Ruby grapefruit	Salto	1997	Stunted	+	+	8	12	12
123	Lemon	San José	1998	Normal	++	+	12	5	10
124	Lemon	San José	1998	Normal	++	UND	11	11	12
125	Lemon	San José	1998	Stunted	+++	-	1	14	8
164	Lemon	San José	1998	Stunted	+++	+	8	12	11
165	Valencia sweet orange	San José	1998	Stunted	-	+	3	13	8
166	Lemon	Canelones	1998	Stunted	+++	-	9	9	10
167	Lemon	Canelones	1998	Normal	++	+	11	11	10
168	Star Ruby grapefruit	Salto	1998	Stunted	-	UND	NC	12	NC
MIL	Satsuma Owari mandarin	Salto	2012	Normal	NA	NA	NC	12	9

References: + mild; ++ intermediate; +++ severe; NA Not Apply; NC Not Cloned; UND Undetermined.

### 4.2. RNA Extraction, cDNA Synthesis, and PCR Amplification

Bark and young leaves of CTV-infected plants were minced into small pieces, frozen at −160 °C for1 h and pulverized in a mortar. Total RNA was extracted using the RNeasy Plant Mini Kit (QIAGEN^®^, Hilden, Germany) following the manufacturer’s instructions. Between 1 ng–5 μg of dsRNA were used as template for cDNA synthesis, and was heat denatured at 65 °C for 5 min in presence of 2 pmol of p25, p20, or p23 gene-specific reverse primer and 1 μL of dNTP Mix (10 mM each) [18,30]. The mixture was transferred to ice for 5 min, and 1 μL of 5× First-strand buffer plus 2 μL of 0.1M DTT was added. After incubation at 42 °C for 2 min was performed, and 200U of SuperScript ™ II Reverse Transcriptase (Invitrogen, Carlsbad, CA, USA) was added. The retrotranscription was performed by incubation at 42 °C for 50 min and an inactivation step at 70 °C for 15 min. For the amplification of the three regions studied, 5 μL of cDNA was PCR amplified in a final volume reaction of 50 μL, with a reaction mix containing 5 μL of 10× Taq Buffer with (NH_4_)_2_SO_4_ (750 mM Tris-HCl (pH 8.8 at 25 °C), 200 mM (NH_4_)_2_SO_4_, 0.1% (*v/v*) Tween 20), 5 μL of dNTP mix (2mM each), 1 μL of each gene-specific primers p25, p20, and p23 (10 μM each) (Table 3), 1.6 μL MgCl_2_ (25 mM), 1.25 U Taq DNA Polymerase Recombinant (Thermo Scientific Inc., Hanover, MD, USA), and nuclease-free water up to 50 μL. The thermal cycling conditions for p20 and p25 genes were as follow: An initial denaturation at 95 °C for 4 min; 35 cycles of 30 s at 95 °C, 30 s at 50 °C, 30 s at 72 °C; and a final extension of 2 min at 72 °C. For the p23 gene, the annealing temperature was 54 °C with the rest of the cycling the same. The resulting amplification products were visualized by GoodView™ (SBS Genetech Co., Beijing, China) staining after electrophoresis in 2% agarose gel. The amplicons were purified using the AxyPrep DNA Gel Extraction kit (Axygen, Corning, NY, USA) following manufacturer’s instructions.

**Table 3:** List of primers used in this work. Nucleotide sequence, genome position, expected size and author reference are described.

Name	Sequence	Genome Position*	Expected Size (bp)	Reference
p20F	5' ACAATATGCGAGCTTACTTTA 3'	17691–17710	555	[18]
p20R	5' AACCTACACGCAAGATGGA 3'	18229–18246		
p23F	5' GTCTCTCCATCTTGCGGTGTAG 3'	18224–18244	733	[30]
p23R	5' CAATCAGATGAAGTGGTG3'	18941–18957		
p25F	5' TGAATTATGGACGACGAAAC 3'	16079–16097	676	
p25R	5' TCAACGTGTGTTGAATTTCCC 3'	16736–16755		

* Respect VT strain (U56902)

### 4.3. Cloning

In order to separate the different genomic variants presumably present in each CTV isolate, RT-PCR purified fragments were cloned with the CloneJET™PCR Cloning kit (Thermo Scientific Inc., Hanover, MD, USA), according to manufacturer’s instructions, followed by transformation of competent *E. coli* NEB5-α cells (New England BioLabs, Hertfordshire, UK). At least ten colonies for sample and gene, containing recombinant plasmids, were examined by PCR. The inserts of the recombinant clones were sequenced in both directions using the pJET1.2 universal primers in an ABI3130 sequencer (Applied Biosystems^®^, Foster City, CA, USA).

### 4.4. Nucleotide Sequences and Phylogenetic Analysis

Consensus sequences were obtained by edition and assembling of both sequenced strands (sense and antisense) for each clone obtained using the SeqMan program from the DNASTAR package [47].

Datasets for each gene were composed of reference strains, Argentinean isolates published by Iglesias and coworkers, different sequences obtained by BLAST from the NCBI database and Uruguayan isolates obtained in this work [30,32]. Based on Harper’s classification system, reference strains for each genotype were retrieved from the GenBank database: U56902 (VT), EU937519 (FS02-2-VT), EU857538 (NZ-M16), JQ965169 (T68), EU076703 (B165), KC525952 (T3), FJ525431 (NZRB-M12), FJ525434 (NZRB-TH30), AF260651 (T30), Y18420 (T385) U16304 (T36), and AY340974 (Qaha) [48]. Datasets were aligned with the MEGA6 software package [33].

The model of nucleotide substitution that best fit each dataset, evaluated by the Akaike Information Criterion (AIC), was selected by jModelTest program [49,50]. The maximum likelihood (ML) method and the GTR model of nucleotide substitution were selected and phylogenies were reconstructed with PhyML in an online web server [51]. Support of the branches was estimated with the approximate likelihood-ratio test (aLRT) with SH-like support. Based on this methodology, three phylogenetic trees were made.

In order to estimate the average distance intra and between genotypes for the three genes, the arithmetic mean of all pairwise distances between two groups in an inter-group comparison was made using MEGA6.

### 4.5. Recombination Analysis

To test whether a recombination event had occurred in any of the sequences included in these studies, a sliding window analysis of distance approach, implemented in the SimPlot program, was used. To find the recombination break points, if necessary, the GARD method from a web server was used.

### 4.6. Nucleotide Sequence Accession Number

All sequences obtained in this work were submitted to the GenBank database under accession numbers KP268157–KP268286 for the p20 gene, KP268287–KP268401 for the p23 gene and KP268402–KP268487 for the p25 gene.

## 5. Conclusions

This study will contribute to a better understanding of CTV population structure in order to perform accurate cross protection programs in our geographic region. The data presented here shows the different genotypes of CTV isolates circulating in Uruguay. The presence of a new genetic lineage reflects the continuous diversification of the virus, reinforcing the importance of deeper genomic studies. Further analysis will be needed, such as biological indexing and full genome sequencing, in order to fully characterize this possible new genotype. The present study intends to open discussion and promote debate in order to achieve a better understanding of the generation and establishment of new CTV genetic variants. Continuous surveillance is important to detect the presence of new CTV genotypes that may cause problems to citrus industries.

## Supplementary Files

Supplementary File 1
