# Supplementary material for: Phylogenetic Studies of the Three RNA Silencing Suppressor Genes of South American CTV Isolates Reveal the Circulation of a Novel Genetic Lineage
Source: Viruses. 2015 Jul 22;7(7):4152–68. doi: 10.3390/v7072814 (PMC4517143; doi:10.3390/v7072814)
Supplement: Supplementary File 1 [file viruses-07-02814-s001.pdf]

Supplementary Materials

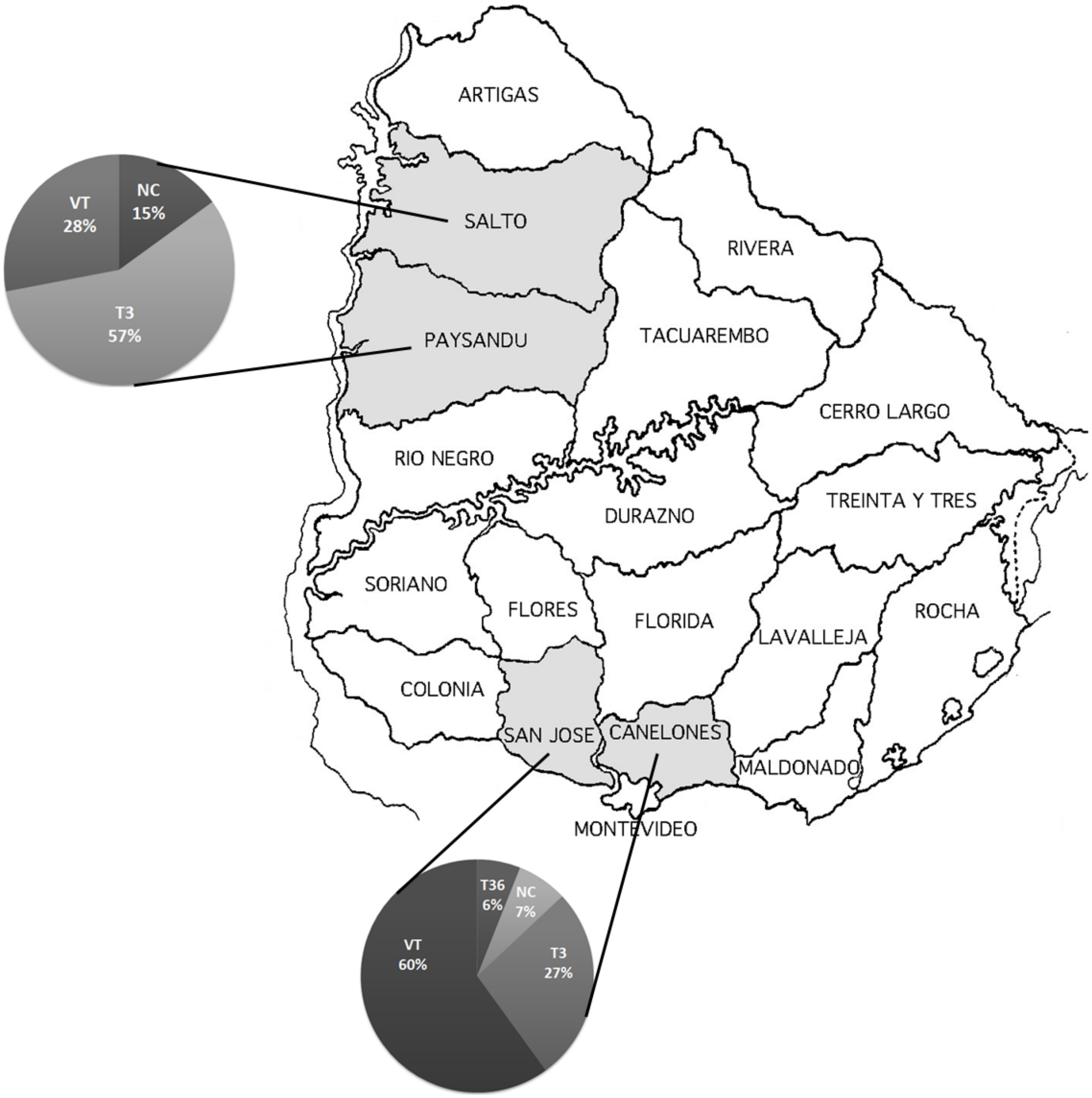

Figure S1. Genotype geographic distribution. Percentage of DNA clones per genotype, for p20 gene in the citrus growing areas of Uruguay.

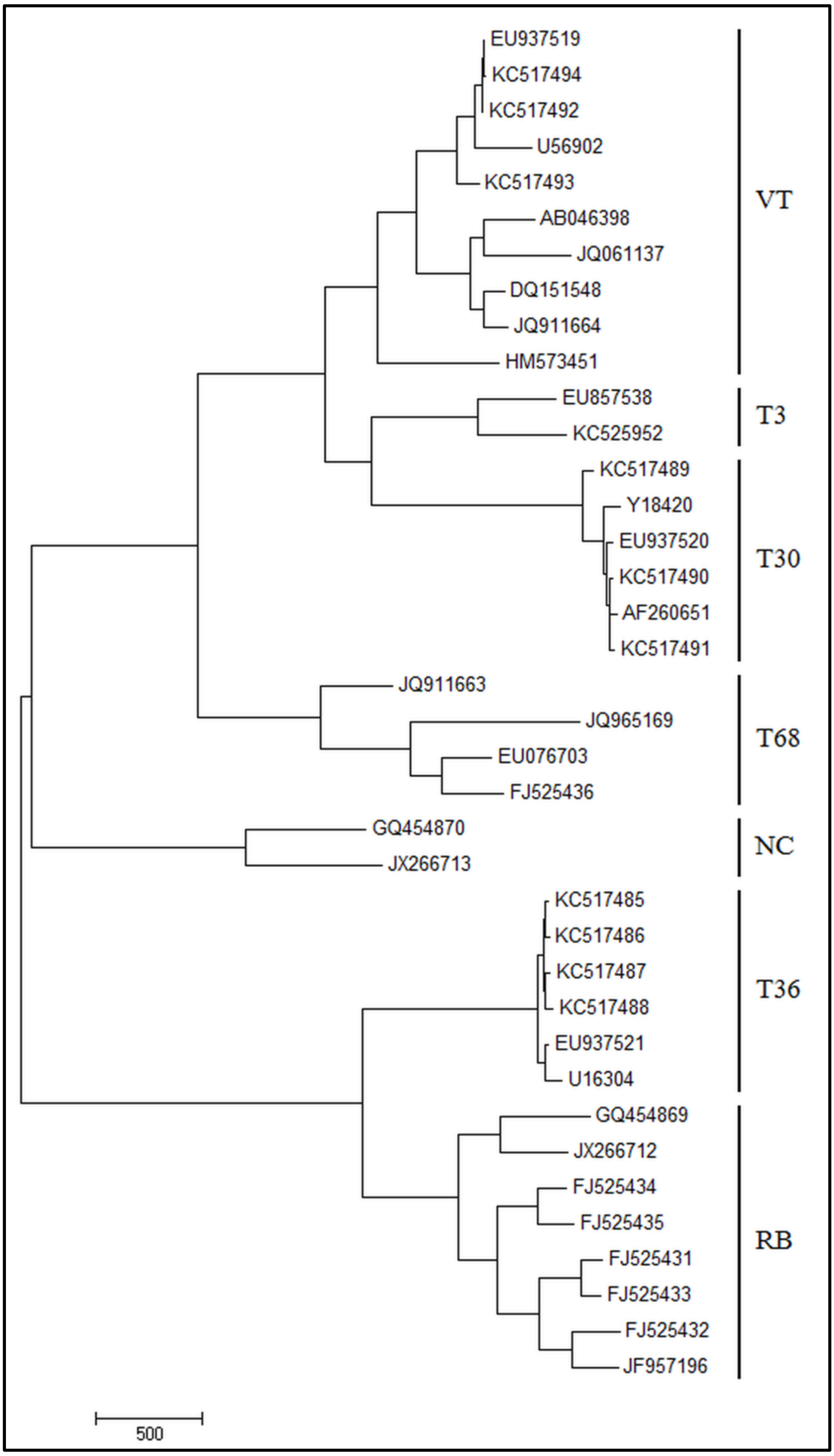

(**A**)

Figure S2.*Cont.*

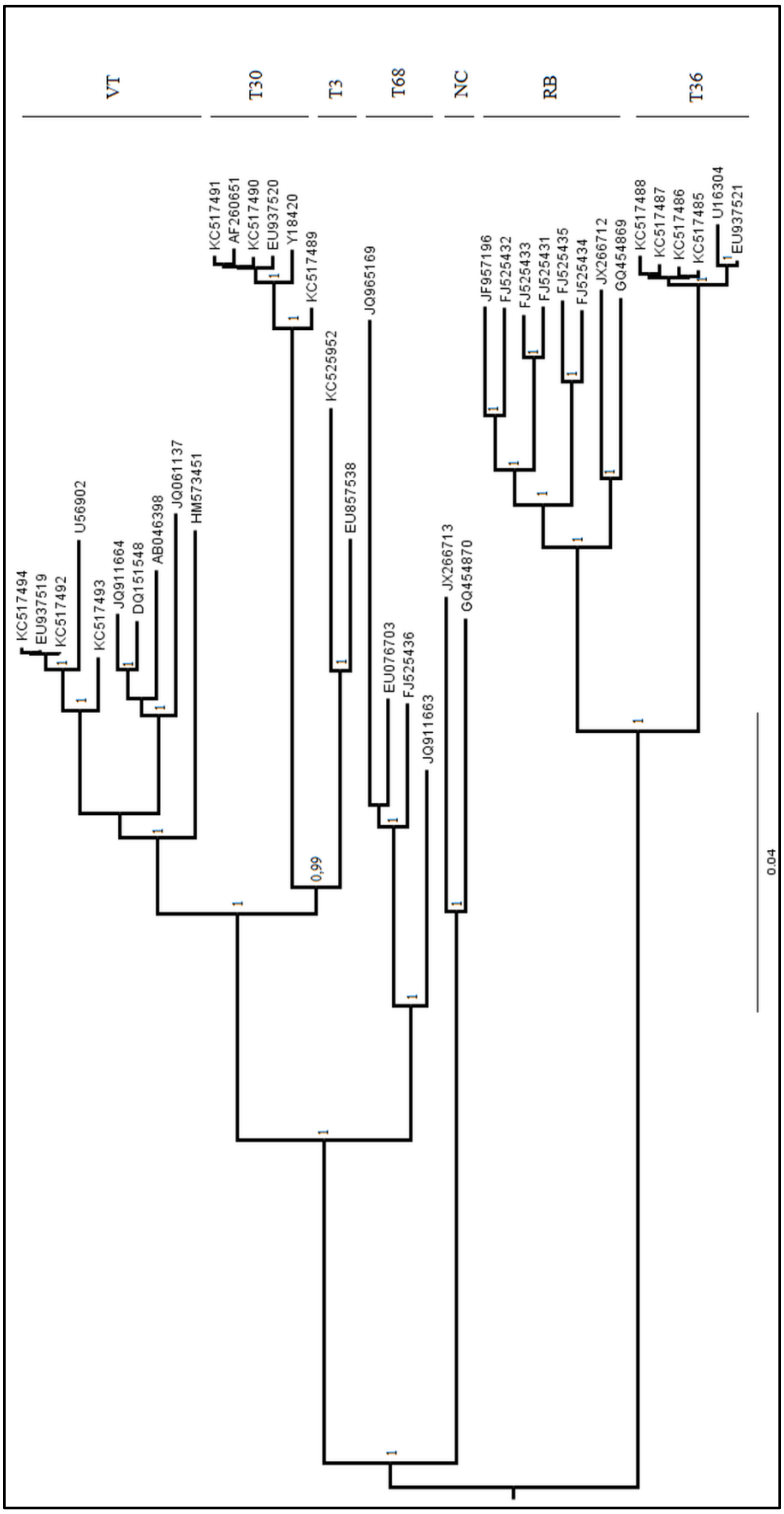

(**B**)

Figure S2. Complete genomes phylogenetic trees of *Citrus Tristeza Virus* isolates. Dataset was composed by isolates from Harper (2013) and Taiwanese strains JX266712-JX266713. (**A**) Maximum Parsimony method was used in the analysis. (**B**) Maximum Likelihood method with HKY nucleotide substitution model and aLRT test to estimate nodes support where used. aLRT values are shown. GenBank accession numbers of strains used are given.
